# The Health Economic Assessment Tool (HEAT) for walking and cycling - experiences from 10 years of application of a health impact assessment tool in policy and practice

**DOI:** 10.3389/fspor.2023.1146761

**Published:** 2023-05-26

**Authors:** Sonja Kahlmeier, Nick Cavill, Meelan Thondoo, Harry Rutter, Thiago Herick de Sa, Francesca Racioppi, Thomas Gotschi

**Affiliations:** ^1^ Department of Health, Campus Zurich, Swiss Distance University of Applied Science (Fernfachhochschule Schweiz FFHS), Zurich, Switzerland; ^2^ Cavill Associates Ltd Bramhall, Stockport, United Kingdom; ^3^ Global Diet and Physical Activity Research Group, Cambridge University, Cambridge, United Kingdom; ^4^ Department of Social & Policy Sciences, University of Bath, Bath, United Kingdom; ^5^ Healthy Urban Environments, Department of Environment, Climate Change and Health, World Health Organization, Geneva, Switzerland; ^6^ European Centre for Environment and Health, WHO Regional Office for Europe, Bonn, Germany; ^7^ College of Design, School of Planning, Public Policy and Management, University of Oregon, Eugene, United States Of America

**Keywords:** economic assessment, health impact assessment, cycling, walking, policy

## Abstract

**Introduction:**

In recent years, walking and cycling have moved into the focus as promising approaches to achieve public health, sustainable transport, climate goals and better urban resilience. However, they are only realistic transport and activity options for a large proportion of the population when they are safe, inclusive and convenient. One way to increase their recognition in transport policy is the inclusion of health impacts of walking and cycling into transport economic appraisals.

**Methods:**

The Health Economic Assessment Tool (HEAT) for walking and cycling calculates: if x people walk or cycle a distance of y on most days, what is the economic value of impacts on premature mortality, taking into account effects of physical activity, air pollution and road fatalities, as well as effects on carbon emissions. Different data sources were collated to examine how the HEAT in more than 10 years of existence, and to identify lessons learned and challenges.

**Results:**

Since its launch in 2009, the HEAT has gained wide recognition as a user friendly, yet robust, evidence-based tool usable by academics, policymakers, and practitioners. Originally designed for use in Europe, it has since been expanded for global use.

**Discussion:**

Challenges for a wider uptake of health-impact assessment (HIA) tools including active transport such as HEAT are the promotion and dissemination to local practitioners and policy makers also outside European and English-speaking regions and in low- and middle-income contexts, further increasing usability, and more generally the advancement of systematic data collection and impact quantification related to walking and cycling.

## 1. Introduction

Over recent decades, a lack of physical activity has been recognized as a major determinant of ill health. Insufficient physical activity has been associated with many chronic diseases, including coronary heart disease, stroke, hypertension, cancer and type II diabetes, as well as excess body weight, poor mental health, and reduced independence in old age. Overall, more than 7% of all-cause and cardiovascular disease deaths and up to 8% of 13 major non-communicable diseases are attributable to insufficient physical activity (1). Globally, almost one out of four adults, and four out of five adolescents are not sufficiently active for good health (2, 3). An increasing number of studies have investigated health impacts from so-called “active travel”, i.e., regular cycling or walking for transport, including combined mobility with public transport. Demonstrated health benefits include a 10% reduction of premature mortality (4–6) and of metabolic risk factors for cardiovascular diseases (5, 6), the prevention of diabetes, some cancers and the reduction of cognitive function in older adults (7) and to some degree lower body weight (8, 9). During the COVID-19 pandemic, cycling and walking have also emerged as travel options that provide physical distancing required by public health measures while at the same time enabling people to remain physically active (10). As a result, many cities around the world have taken steps to promote walking and cycling (11, 12).

In recent years transport-related physical activity has moved into focus as a promising approach to reach parts of the population for whom sport is not amenable, attractive or affordable (13, 14). Both the “Eigth Investments that work” (15) and the Global Action Plan on Physical Activity include actions related to promoting safe, active transport (16). In 2021, the first European Masterplan for Cycling was adopted (17) and a new plan for walking promotion is under development. There is merit in increasing collaboration between the transport and health sectors to achieve transport goals such as reducing congestion or climate change (18, 19) as well as public health targets to reduce physical inactivity and disease burden (14, 20, 21). For example, in England and Wales, on average only 10 min per day are spent walking for transport, and less than 2 min for cycling. That more is possible beyond champion countries such as the Denmark or the Netherlands is seen in Switzerland, where over 20 min are spent for transport-related walking and 4.5 min for cycling each day (21). Such differences in transport behaviour translate into considerable public health impacts (14, 20, 21). In addition, evidence from 30 studies from Europe, as well as Australia, New Zealand and the United States of America shows that the benefits from physical activity clearly outweigh potential harm from air pollution or traffic crashes (22). A similar pattern is seen in evidence from some low- and middle-income settings, such as Brazil (23), Mauritius (24) or India (25).

For walking and cycling to present realistic transport alternatives for a large share of the population, they need to be safe and convenient (11, 13, 14, 16). Planning practice can draw from a wide portfolio of well-established infrastructure and traffic regulation measures to make active travel modes more palatable. However, the priority given to the promotion of active travel modes in transport planning varies widely, as differences exist between countries, cities, towns, and rural areas (18, 26). In addition, issues of inequity and social justice influence the relationship between transport and health by modifying the exposure and severity of health effects and outcomes (27, 28), further stressing the need to estimate variations within and across local populations.

Quantifying the potentially substantial benefits of active travel measures has been recognized early on as a promising approach to elevate the status of walking and cycling within the planning as well as the public health community (29–31). Cost-benefit analysis is a standard methodology in transport planning: in many countries, a road, bridge or cycle path will not be built unless the benefits can be shown to be greater than the costs. However, traditionally in many cases walking and cycling have not been included in economic valuation of transport projects (31–33), putting active forms of travel at a relative disadvantage since the full benefits are not considered. Thus, providing methodological approaches to include the benefits of active travel is one approach to moving walking and cycling up the transport policy agenda.

In response, in 2005 a multi-phase, open-ended project was established to develop the Health Economic Assessment Tool (HEAT) for walking and cycling as a harmonized method for economic valuation of health impacts of walking and cycling, based on best available evidence and international expert consensus (34, 35). Since then, implementation has been steered by a core project group, working in close collaboration with advisory groups, led by the WHO Regional Office for Europe and Headquarters.

The HEAT calculates: if x people cycle or walk y distance on most days, what is the economic value of resulting reductions in mortality? This calculation can serve different types of assessment, for example, of current (or past) levels of cycling or walking, such as showing the value of cycling or walking in a city or country; of changes over time, such as comparing before-and-after situations or scenario A vs. scenario B (such as with or without measures taken); and evaluating new or existing projects, including calculating benefit–cost ratios. The tool is intended to be robust but easy to use primarily by transport planners, traffic engineers, economists and special interest groups.

In 2007, an approach to calculating the economic value of reduced all-cause mortality from cycling (quantified using the Value of a Statistical Life VSL approach) was adopted at the first HEAT consensus meeting (34, 35). These international consensus meetings are a core part of the HEAT development to promote critical review, discussion and achievement of consensus on proposed options, including in cases where best expert judgement is needed (35). The HEAT process follows the following key principles:

• Robust and evidence-based Decisions are taken on the best available evidence with the view of providing a scientifically robust tool.
• Easily usable The HEAT is developed for the transport sector as the main target audience, thus for users who are likely to have limited knowledge of epidemiology and public health. Simplicity in application is thus a key consideration for any decision taken.
• Transparent
  Decisions and any assumptions taken are made transparent.
• Conservative
  Methodological considerations are taken with an “at-least” approach.
• Adaptable
  While for as many as possible inputs, default values are provided to facilitate application, which in most cases can be adapted to reflect local conditions and approaches.
• Modular
  Users can select to run assessments for different modes of transport (i.e. walking, cycling and soon e-biking) as well as different impact pathways (physical activity, air pollution, fatal traffic crashes, and carbon emissions). In addition, HEAT is meant for integration into wider cost-benefit analysis as a modular component.

A key feature of the HEAT process is the involvement of experts from a range of fields that are relevant for the development and implementation of walking and cycling measures. To date, next to the coordinating team, more than 120 experts from epidemiology, public health, transport research and planning, health and transport economics, air pollution and environmental sciences, policy making, practice and advocacy from over 30 countries have contributed to the development of the HEAT across the different project phases.

Based on a systematic review of the literature (33), a basic approach designed for the European region was agreed to develop the first HEAT for cycling (36, 37), launched in 2009. An updated version of the HEAT for cycling and a new HEAT for walking were finalized at a second consensus meeting and launched in 2011 as a web-based tool (34) with a methods and user guide booklet. In 2013, the third consensus meeting discussed results from another systematic review (4) to update the methods for HEAT walking. A new HEAT version including an option to calculate health impacts from air pollution and road fatalities separately, and to take into account carbon effects of shifting to active modes was launched in 2017 (38, 39). In 2021, the first globally applicable version was presented (40). Future versions are expected to include e-biking and translations into different languages.

This paper discusses how the HEAT has been used over its more than 10 years of existence, and identifies lessons learned and challenges for the ways in which economic assessments of their health impacts can foster the recognition of walking and cycling in transport policy and practice.

## 2. Methods and materials

To collect information on the uptake of HEAT, a range of sources has been used:

• Applications saved in the HEAT online database by users between 2011 and 2015,
• Applications communicated to the HEAT coordinating team by email,
• Collections of applications gathered on behalf of the WHO Regional Office for Europe in 2012 and in 2021,
• Invitations to report applications to the HEAT mailing lists sent from the WHO Regional Office for Europe in 2015 (41),
• A systematic online search using the Google search machine and the PubMed literature database, carried out in summer 2015, and updated searches in 2017 and 2021 (42), using also Google Scholar,
• Responses to a questionnaire on the monitoring framework for the implementation of policies to promote health-enhancing physical activity in the EU and the WHO European Region (containing one question on HEAT) returned in April 2015 (43), and
• The reference lists of 3 systematic reviews on health impact assessments of active travel (22, 44, 45).

In addition, in 2015 an online survey with 8 questions on the experiences with using the HEAT was carried out (41). The survey focused on general use, applicability, and challenges around the HEAT, thus it is still of relevance even if tool functionalities have changed since then. Invitations were sent by email to 2,865 HEAT users known from the above-listed sources and other stakeholders, including the Transport, Health and Environment Pan-European Programme (THE PEP) (46) and the European network for the promotion of health-enhancing physical activity (HEPA Europe) (47). 263 responses were received (9% of the total sample) (41). Finally, 11 semi-structured interviews were carried out with selected HEAT users in 2015 to understand in more detail how HEAT had been used and possibly influenced transport policy and practice.

Web statistics on the HEAT use are available since its launch as an online tool in May 2011 through the Google Analytics tool (48); however, due to migration of HEAT to a new technical platform in 2017, long term trends are not fully comparable. The metric “non-bounce users” excludes users who had no interaction with the page (i.e., just opening, looking, closing), available as of November 2020.

## 3. Results

### 3.1. Overview of dissemination and uptake

Since the launch of the first online version in May 2011, the tool had over 544,000 page views by over 40,600 users. While originally developed for the WHO European Region, the HEAT use was widespread beyond Europe, with the top countries in the last 2 years (November 2020–2022) being the United Kingdom and the United States, followed by China, Germany, France, Italy, and Finland, Spain, Switzerland and Australia, in addition the to use of the tool by cities such as London, Shanghai, Helsinki, Vienna and Paris.

While HEAT was developed primarily for the transport sector, the majority of online survey respondents of 2015 came from the public health sector (43%), with similar shares of responses from transport (28%) and academic sectors (27%) (41). Twenty-two percent of survey respondents had not heard of or looked at the HEAT, 44% had at most entered some data to see how it worked. As main reasons for not using the HEAT, lack of time (41%) or lack of suitable data (38%) were quoted. About one third of respondents (*n* = 78, 31%) had done one or more full calculations (34). Of these, 47% estimated the value of future projected or hypothetical levels of cycling or walking, 22% the value of measured increases and 19% that of current levels of cycling or walking. Results were most often used for a presentation (39%), internal (37%) or published (27%) reports or an academic paper (15%). Main target audiences were a local authority or municipality (59%), a national authority/ministry (27%), a research body (23%) or a non-government organization (21%).

The data from the different information sources (see Methods and materials) yielded a total of 132 documented applications (41, 42), including 7 that mentioned HEAT but did not apply it in practice or were incomplete draft reports.

The remaining 125 applications included 52 technical reports and 38 academic publications, as shown in Table 1, as well as 12 government papers or guidance reports. Twelve of the publications qualified as government papers or guidance, i.e., documents issued by a part of an administration and/or guidance documents that promote the use of HEAT. There were also several reports from academic institutions or consultancies that were developed on behalf of administrative bodies, as well as research reports, in particular by local administrations.

**Table 1:** Documented applications of the Health Economic Assessment Tools (HEAT), by type.

Type	Number	Percent	Examples
Reports	52	41.6%	
*English*	32	25.6%	(49–51)
*Non-English*	20	16.0%	(52–54)
Academic paper/abstract	38	30.4%	(55–57)
Government papers/guidance	12	9.6%	(58–60)
Other (case studies, slides, website etc.)	23	18.4%	(61, 62)
Total	125	100%	

Despite the wide-ranging searches and repeated invitations to report applications, in comparison to the web statistics, to date there is still only a limited number of documented uses of HEAT by government agencies, and a majority of those come from the United Kingdom. This may be due to an English language bias, as the HEAT has only been available in English (despite user guides having also been translated into German, French, Finnish, Polish, and Spanish), likely to reflect the differential uptake of the HEAT by certain countries. In addition, there is only limited evidence on the tool's direct impact on policy actions and decision-making.

While the HEAT has not been developed as a research tool, usage in almost 40 academic publications confirms its scientific robustness and usefulness in an academic setting, including for training purposes.

In the 11 interviews carried out in 2015 with users (41), a number of strengths and weaknesses of the HEAT were identified (see Table 2).

**Table 2:** Strengths and weaknesses of the Health Economic Assessment Tools (HEAT) for walking or cycling.

Strengths	– Allows quantifying the magnitude of the health impacts compared to other (e.g., environmental) benefits. – Combination of health impacts and economic quantification. – Having a specific number gets people thinking and facilitates discussions and exchange with relevant bodies. – Evidence- and consensus-based approach, transparency. – Approach allows integration into existing transport appraisal systems. – Can facilitate the exchange between transport and health specialists, where a desire exists. – Tool now also adapted for global use. – Project coordination by the WHO adds to credibility.
Weaknesses	– In some audiences the use of Value of a Statistical Life and resulting numbers are not easily understood. – HEAT is not well known in local communities where many transport planning decisions take place. – Translations of the English tool and user guide into local languages are needed for uptake, in particular on the local level. – As long as it is not included in the official national guidance for transport appraisals, it will only be used seldom.

### 3.2. Examples of applications

In this section we present selected examples of HEAT applications from academia, policy and practice.

#### 3.2.1. Academia

##### 3.2.1.1. An economic analysis of four Ciclovía programs

The Ciclovía is a regular multisectorial community-based program in which streets are temporarily closed for motorized transport, allowing exclusive access to individuals for recreational activities and physical activity. In this early application of the HEAT, a cost–benefit analysis of physical activity of the Ciclovía programs of Bogotá and Medellín in Colombia, Guadalajara in México, and San Francisco in the United States was carried out (55). The study found that the cost–benefit ratio for health benefit from physical activity was highly positive, ranging from 3.23–4.26 for Bogotá, 1.83 for Medellín, 1.02–1.23 for Guadalajara, and 2.32 for San Francisco.

##### 3.2.1.2. Exploring the health and spatial equity implications of the New York city bike share system

In this study, the HEAT was part of the assessment scheme of the New York Citi Bike share system benefit at launch in 2013 and after expansion in 2015 (56). The study also discussed how further system expansion and utilization by residents in high-poverty communities could affect the potential benefit of the largest bicycle share system in the United States. The results showed that the greatest proportion of Citi Bike stations were located in low-poverty (i.e., wealthier) census tracts (41% per period), and there were no significant changes in station distribution during expansion. HEAT estimated an increase from two to three premature deaths prevented and an increased annual economic benefit from $18,800,000 to $28,300,000 associated with Citi Bike use. The findings underlined the potential for even greater benefits with advancing access in higher-poverty neighbourhoods and communities of colour.

#### 3.2.2. Policy: Austrian Masterplan walking and cycling

The Federal Ministry for Climate Action, Environment, Energy, Mobility, Innovation and Technology has been involved in the development of HEAT since the very beginning and sponsored the first HEAT consensus meeting in Graz in May 2007. HEAT is currently promoted in three ways: they translated HEAT and the user guide into German when it was launched as an online tool in 2009 and they make HEAT available on the ministries' website (61). They also calculated the societal economic value of health effects from current and levels and aspired policy goals for cycling in Austria using the HEAT in 2009 and again in 2014 (62); and they included a mention of the HEAT into the National Masterplan for Cycling (p. 43) (58). The HEAT results have also been used regularly in presentation and communications of the ministry, e.g., in relation to a cycling tour of the former Minister of Environment.

#### 3.2.3. Practice: health impact assessment of fare increases and service cuts on public transport (Boston, Massachusetts, Uniteed States)

The Metropolitan Area Planning Council used the HEAT as part of a rapid health impact assessment to calculate the economic costs of potential increases in mortality as a consequence of decreases in regular walking due to two proposals to increase fares and cut services on public transport (63). Results showed that across the two proposals there would be 9–14 additional deaths per year due to decreased physical activity, which could be valued at between $74.9 and $116.5 million per year. This significant cost associated with the proposed fare and service changes was second only to the cost of time lost to congestion. The resulting costs would have by far exceeded the budget shortfall that the proposed changes sought to address. According to the authors, the results contributed to lower fare increases and fewer service cuts than initially proposed.

## 4. Discussion

With the growing popularity of walking and cycling as healthy and sustainable travel modes, over the past decade, economic assessments of health impacts from walking and cycling have become more established, thanks to practical yet robust tools such as the HEAT or the Integrated Transport and Health Impact Modelling Tool (ITHIM) (64). The large number of officially published applications by academia, governments, NGOs and consultancies demonstrates a wide recognition of the HEAT as an established and valued tool to calculate health and carbon impacts from active transport interventions. It was also found to be the most widely used health-impact assessment tool for active travel by two systematic literature reviews (44, 45). Important milestones of this progress have been the endorsement of the HEAT in the Global Action Plan for Physical Activity (p. 69) (16), and most recently in the Pan-European Cycling Masterplan (17).

### 4.1. Success factors and achievements of HEAT

HEAT has been well-received and often characterized as an “eye-opening” tool by a broad audience of transport and health advocates, both within and outside of governmental institutions. This is due to monetization of health benefits using the value of statistical life approach results in benefit estimates which exceed general expectations. In some occasions, this plain quantification of impacts in monetary terms has earned active travel modes “a seat at the table” (41), resulting in more equal consideration of walking and cycling in decision-making processes. While examples of a direct impact on policy decisions remain rare, HEAT results have equally resonated in places with low levels of walking or cycling to make a basic case for their benefits, as well as in places with well-established walking or cycling cultures to make more nuanced benefit-vs.-cost arguments. This is particularly the case in countries where economic appraisal is an established practice, such as the United Kingdom: here, the HEAT has become very established because there is a strong tradition of putting transport proposals through an economic analysis (41, 59). HEAT also has proven helpful to put benefits from physical activity in perspective to harms from air pollution and insufficient traffic safety while walking or cycling. While research has shown that in general benefits of active travel outweigh the risks (22, 65, 66), HEAT provides users with the possibility to verify or challenge this assumption for their particular local case for example by comparing impacts in a more and a less cycling friendly city (67), helping to counteract unbased fears or to substantiating calls for safety improvements (68). The recognition of the potential role for active travel in efforts to reduce urban carbon emissions (19) further increases the usefulness of the HEAT, supporting the inclusion of this argument into appraisals of different policy options.

Being steered by the WHO and making co-creation with leading experts from around the world part of its key approach, the HEAT has earned a status of credibility and scientific robustness by academics, advocates and governments alike. Two national agencies, namely in England and Austria, have officially endorsed the HEAT at some point (41).

Arguably the HEAT tool's greatest success factor is to present the impact assessment as a short sequence of steps, when in fact the calculation needs to rely on a fairly complex set of data, methods and assumptions (36, 38, 40). Throughout its evolution, the HEAT has made it a top priority to require minimally inputs to address user needs, while providing as much of the required data and calculations as possible in the background. In the simplest cases, a handful of inputs is sufficient to obtain results. At the same time, over the years the tool has evolved to accommodate both a more diverse set of applications as well as increasingly diverse audiences and levels of expertise.

### 4.2. Limitations and challenges of HEAT

Despite its considerable success, substantial challenges remain for HEAT, many of which apply to HIA tools for active transport in general. The most common difficulty reported by HEAT users is how to obtain data on active travel use. By design, HEAT leaves the burden of assessing or estimating levels of walking and cycling to its users. While in some circumstances hypothetical calculations based on (often crude) scenario assumptions are sufficient, many users desire to quantify concrete impacts of existing or planned programs or infrastructure projects. However, in particular for small scale projects assessing walking and cycling levels accurately and predicting effects of interventions on future active travel use remains a challenge. Although data collection is outside the immediate scope of HEAT, there is a role for the HEAT project to guide users through the ever-evolving methods for active travel estimation, as the institutional burden to use online surveys or app-based tracking becomes smaller over time.

A limitation of HEAT is that health impacts are assessed based on mortality risk (i.e., premature deaths and road fatalities) only. Although there is ample evidence aor impacts of active travel on morbidities (5–9), the methodological implications of including these in tools such as HEAT have to date presented to be at odds with its main goal of providing a simple-to-use tool. Thus, health impact estimates derived by HEAT present relatively crude ballpark estimates and do not allow specific conclusions regarding health outcomes such as mental health or cardiovascular disease.

HEAT uses the VSL to quantify the societal value of reductions in mortality risk. The values are widely available (69), and recently a methodology was integrated into the HEAT tool to estimate VSL estimates for each country worldwide (38, 40). While widely used in transport appraisals and elsewhere, monetization based on this approach does not provide estimates in terms of health care savings or other governmental expenditures, and as such remains hard to explain, particularly non-transport experts and some local decision-makers.

### 4.3. Outlook

The primary goal of HEAT and other HIA tools for active travel modes is to provide decision-making processes that affect walking and cycling with robust, quantitative inputs. This goal has been pursued through a three-pronged strategy: (a) provide a scientifically robust tool broadly accepted by experts and governmental institutions, (b) provide a simple-to-use tool easily picked up by practitioners within and outside of governmental institutions, and (c) disseminate the tool and related success stories as widely as possible. While tool development remains ongoing (e.g., integration. Of e-biking), with the launch of the globally applicable version the priority now will shift towards wider dissemination.

As with many innovations, successful adoption of the HEAT has been greatly helped by enthusiastic “early adopters” who see its potential and make the effort to try it out and then advocate for it. More strategically developing and supporting networks of such advocates will be a key task for building capacity and increasing the use of the tool in the future. The challenge is identifying the right people, getting them to spend time to apply it to a specific scenario or case study and to promote it at a national and/or local level.

Continued strategic communication, capacity building and dissemination is thus another key task for the future, along with translation of the tool, user guide and the website, particularly into languages making it amenable outside of Europe, e.g., into Portuguese or Spanish. Another element is to collate more case studies that are applicable to low- and middle-income contexts supporting a more global uptake of the HEAT, particularly considering the recent adaptation of the tool for global use.

Finally, lack of systematically collected data on walking and cycling was identified as a key barrier to use of HIA tools such as the HEAT. Users stated that trying the HEAT often had an unexpected outcome of helping them realise the gaps in their data. Supporting international, national and sub-national transport authorities in collecting better data on walking and cycling should be another key component of a future HEAT dissemination strategy.

In conclusion, the contribution of walking and cycling to addressing public health, sustainable transport and climate goals are substantial, particularly in settings facing increasing levels of urbanisation and motorisation. Therefore, integrating health impacts into transport economic assessments should be a standard approach for all transport planning approaches to ensure that the most cost-effective and thus sustainable investments are made.

## Data Availability

The raw data supporting the conclusions of this article will be made available by the authors, without undue reservation.
